# Calcitonin gene-related peptide promotes mast cell-mediated neuroimmune inflammation through the CALCRL/RAMP1–JAK3–STAT1 axis in rosacea

**DOI:** 10.3389/fimmu.2026.1930591

**Published:** 2026-09-02

**Authors:** Xiaojin Li, Huiping Fan, Rui Sun, Qingsong Ma, Jiayun Liu, Chengqi Liu, Dong Zhang, Weiyuan Ma

**Affiliations:** Department of Dermatology, Affiliated Hospital of Shandong Second Medical University, School of Clinical Medicine, Shandong Second Medical University, Weifang, Shandong, China

**Keywords:** calcitonin gene-related peptide, CALCRL/RAMP1, JAK–STAT signaling, mast cells, neuroimmune inflammation, rosacea

## Abstract

**Introduction:**

Rosacea is a chronic inflammatory skin disorder characterized by neurovascular instability and dysregulated innate immunity. Although mast-cell activation is increasingly recognized as a central pathogenic feature, the neuroimmune mechanisms linking neuropeptide signaling to mast cell-mediated inflammation remain incompletely defined. This study investigated whether calcitonin gene-related peptide (CGRP) promotes mast cell-mediated inflammation in rosacea and explored the underlying signaling mechanism.

**Methods:**

Bioinformatic analyses of the GSE65914 dataset were performed to characterize rosacea-associated molecular pathways and mast-cell signatures. Serum and skin samples from patients with rosacea and healthy controls were analyzed by ELISA, histological staining, and immunofluorescence. *In vitro*, CGRP-induced mast-cell activation and CALCRL/RAMP1–JAK3–STAT1 signaling were investigated in LUVA cells using RT-qPCR, western blotting, immunofluorescence, degranulation assays, ELISA, and pharmacological approaches, while the potential CALCRL–JAK3 association was assessed by molecular docking and reciprocal co-immunoprecipitation. *In vivo*, ACK2-mediated mast-cell depletion and pharmacological blockade of the CGRP receptor or JAK3 were evaluated in CGRP-aggravated LL37-induced rosacea-like mouse models.

**Results:**

Rosacea lesions showed enrichment of immune-inflammatory pathways, JAK–STAT signaling, and mast cell-associated signatures. Serum CGRP levels were elevated in patients with rosacea and positively correlated with flushing and erythema severity. Lesional skin showed increased CGRP expression and enhanced localization of CALCRL, RAMP1, phosphorylated JAK3, and phosphorylated STAT1 in dermal CD117+ mast cells. In LUVA mast cells, CGRP upregulated CALCRL/RAMP1 expression, activated JAK3–STAT1 signaling, and promoted degranulation, histamine release, and inflammatory mediator production. Molecular docking and reciprocal co-immunoprecipitation supported a potential association between CALCRL and JAK3. Rimegepant or ritlecitinib attenuated CGRP-induced JAK3–STAT1 activation and mast-cell responses. Modulation of the canonical Gαs–cAMP–PKA pathway did not abolish CGRP-induced JAK3–STAT1 phosphorylation, indicating that this response is not primarily mediated by the cAMP–PKA cascade. *In vivo*, mast-cell lineage depletion attenuated CGRP-driven exacerbation of LL37-induced rosacea-like inflammation, while rimegepant or ritlecitinib alleviated inflammatory changes and reduced JAK3–STAT1 activation and mast-cell responses.

**Conclusion:**

These findings identify a CGRP-associated neuroimmune pathway in rosacea in which CALCRL/RAMP1-linked JAK3–STAT1 signaling promotes mast cell-mediated inflammatory amplification. Targeting the CGRP–CALCRL/RAMP1–JAK3–STAT1 axis may provide a mechanism-based therapeutic strategy for neurovascular-dominant or treatment-refractory rosacea.

## 1 Introduction

Rosacea is a chronic inflammatory disorder of the central face characterized by recurrent flushing, persistent erythema, telangiectasia, and papulopustular lesions. These cutaneous manifestations are often accompanied by burning, stinging, heightened sensory reactivity, and a substantial psychosocial burden (1, 2). Although current therapies, including oral antibiotics, isotretinoin, topical anti-inflammatory agents, and vascular-targeted interventions, can provide symptomatic improvement in selected patients, treatment responses remain heterogeneous and recurrence is common (3). A deeper understanding of the upstream mechanisms that integrate neurovascular dysregulation with cutaneous immune activation is therefore needed to support the development of mechanism-based therapeutic strategies.

Mast cells have emerged as important amplifiers of rosacea-associated inflammation (4–6). Anatomically, mast cells are enriched at neurovascular–immune interfaces, where they are positioned to sense mediators released from sensory nerves, endothelial cells, keratinocytes, and innate immune pathways (7, 8). Functionally, activated mast cells release a broad range of inflammatory and vasoactive mediators, including cytokines, chemokines, proteases, histamine, and angiogenic factors, which can promote vasodilation, vascular permeability, leukocyte recruitment, and tissue remodeling (9–11). Experimental studies have shown that LL37, an antimicrobial peptide strongly implicated in rosacea pathogenesis, fails to induce robust rosacea-like skin inflammation in mast cell-deficient mice, highlighting the essential role of mast cells in disease initiation and amplification (4, 12). However, the upstream neuroimmune signals that drive mast-cell activation in rosacea remain incompletely defined.

Neurogenic inflammation is increasingly recognized as a central pathogenic component of rosacea (13–15). Calcitonin gene-related peptide (CGRP), a potent vasoactive neuropeptide that can be released from cutaneous sensory nerves, has been implicated in facial flushing, neurovascular instability, and inflammatory responses (16, 17). Elevated circulating CGRP levels have been reported in patients with rosacea, and emerging clinical observations suggest that blockade of CGRP signaling may improve flushing and persistent erythema in selected patients (18–20). Given the close anatomical association between sensory nerve fibers and dermal mast cells, and the potential contribution of CGRP from both neuronal and non-neuronal sources, CGRP represents a plausible upstream mediator linking neuroimmune signaling to mast cell-mediated inflammation (15, 21, 22). Nevertheless, whether CGRP directly activates mast cells in rosacea, and which intracellular signaling pathways mediate this response, remain unclear.

CGRP classically signals through a heterodimeric receptor complex composed of calcitonin receptor-like receptor (CALCRL) and receptor activity-modifying protein 1 (RAMP1), leading to activation of the Gαs–adenylyl cyclase–cAMP–protein kinase A (PKA) pathway (23, 24). Although this canonical pathway is well established in neurovascular signaling, CGRP may also exert context-dependent immunomodulatory effects that vary according to cell type, tissue microenvironment, and disease state (25, 26). In parallel, the JAK–STAT pathway has gained increasing attention in rosacea pathophysiology. Enhanced JAK–STAT activity has been detected in rosacea lesions, and pharmacological inhibition of JAK-associated pathways has shown therapeutic potential in inflammatory skin disease (27–30). JAK–STAT signaling is also involved in mast-cell maturation, activation, and cytokine production (31). However, whether CGRP receptor activation can engage non-canonical JAK–STAT signaling in mast cells has not been explored.

In this study, we identify a previously underrecognized CGRP–CALCRL/RAMP1–JAK3–STAT1 neuroimmune axis that contributes to mast cell-mediated inflammation in rosacea. By integrating transcriptomic analysis, clinical sample validation, lesional immunofluorescence, *in vitro* mast-cell experiments, protein-interaction assays, mast-cell depletion, and pharmacological intervention in rosacea-like mouse models, we show that CGRP receptor signaling is associated with JAK3–STAT1 activation in dermal mast cells. We further demonstrate that CGRP promotes mast-cell degranulation and inflammatory mediator release, while CGRP-induced JAK3–STAT1 activation is not primarily mediated by the canonical cAMP–PKA cascade. Finally, we show that targeting CGRP receptor signaling with rimegepant or inhibiting JAK3 with ritlecitinib attenuates mast-cell activation and alleviates rosacea-like dermatitis induced by combined CGRP and LL37 in mice.

Collectively, our findings support a role for CGRP as a neuroimmune amplifier in rosacea by linking elevated CGRP signaling to mast cell-mediated inflammation. This pathway integrates neurovascular instability, mast-cell activation, and inflammatory mediator production within a unified pathogenic framework and suggests that the CGRP–CALCRL/RAMP1–JAK3–STAT1 axis may represent a potential therapeutic target for neurovascular-dominant or treatment-refractory rosacea.

## 2 Materials and methods

### 2.1 Clinical samples

Serum samples were collected from 38 patients with rosacea and 32 age-matched healthy controls aged 20–50 years. Mid-facial skin biopsies were obtained from five patients with rosacea and five age-matched healthy volunteers undergoing elective plastic surgery. Flushing severity, erythema severity, and overall disease severity were evaluated using the Global Flushing Severity Score (GFSS), Clinician’s Erythema Assessment (CEA), and Investigator’s Global Assessment (IGA), respectively (32, 33).

The use of all human samples was approved by the Ethics Review Committee of Shandong Second Medical University (Approval No. SDSMU-2025-ky-228). Written informed consent was obtained from all participants. The study was conducted in accordance with the Declaration of Helsinki, local legislation, and institutional requirements.

### 2.2 Cell culture and treatments

The human mast cell line LUVA (Cat. No. CTCC-001-0351) was purchased from Zhejiang Meisen Cell Technology Co., Ltd. and cultured in StemPro-34 SFM medium supplemented with 1× GlutaMAX. Human embryonic kidney 293T cells (HEK293T; Cat. No. CL-0005) were purchased from Wuhan PunoSai Life Science Technology Co., Ltd. and cultured in DMEM containing 10% fetal bovine serum (Sangon Biotech, Shanghai, China). Both cell lines were maintained at 37 °C in a humidified incubator containing 5% CO_2_.

For *in vitro* experiments, human α-calcitonin gene-related peptide (α-CGRP; 37 aa; ACDTATCVTHRLAGLLSRSGGVVKNNFVPTNVGSKAF-NH_2_) was custom synthesized by Sangon Biotech (Shanghai, China), with a purity of >95% verified by high-performance liquid chromatography (HPLC). For *in vivo* experiments, rat α-CGRP (GenScript, Cat. No. RP11095; purity >95%) was used. Both peptides were dissolved in PBS and stored at −80 °C until use. For CGRP stimulation, LUVA cells were treated with 100 nM CGRP diluted in sterile PBS for the indicated times. For mast-cell degranulation assays, LUVA cells were stimulated with CGRP for 30 min before supernatants and cell lysates were collected. For pharmacological intervention experiments, LUVA cells were pretreated with rimegepant (1 μM; Selleck, S6659), ritlecitinib (500 nM; Selleck, S8538), fludarabine (10 μM; Selleck, S1491), suramin (100 μM; Selleck, S8942), or forskolin (FSK; 10 μM; Selleck, S2449) for 30 min, followed by incubation with or without CGRP for the indicated durations. Rimegepant, ritlecitinib, fludarabine, suramin, and FSK were dissolved in DMSO, and the final DMSO concentration was kept identical across all treatment groups. Cells and culture supernatants were collected for subsequent analyses.

HEK293T cells were transiently transfected with the indicated single plasmids or co-transfected with plasmid combinations. FLAG-CALCRL and HA-JAK3 overexpression plasmids were transfected using TurboFect transfection reagent (Thermo Fisher Scientific, R0531) according to the manufacturer’s protocol. Gene overexpression efficiency was confirmed by RT-qPCR and western blotting. All overexpression plasmids used in this study were synthesized by Jinan Boshang Biotechnology Co., Ltd.

### 2.3 Mice

All animal experiments were approved by the Animal Ethics Committee of Shandong Second Medical University (Approval No. 2025SDL756). Eight-week-old female BALB/c mice and eight-week-old female C57BL/6 mice were obtained from Beijing Vital River Co., Ltd. A total of 36 BALB/c mice and 30 C57BL/6 mice were used in this study. The animals were acclimatized for 1 week before experiments and housed under controlled environmental conditions at 22 °C with a 12-h light/dark cycle and free access to food and water.

### 2.4 Induction of rosacea-like dermatitis and drug administration

The CGRP-aggravated LL37-induced rosacea-like dermatitis model was established according to a previously described protocol (13). LL37 peptide (Sangon Biotech; purity ≥95%) was dissolved in sterile PBS at a concentration of 320 μM. CGRP (GenScript) was dissolved in sterile PBS to prepare a working concentration of 1 μM. A mixed working solution containing 320 μM LL37 and 1 μM CGRP was also prepared.

Eight-week-old female BALB/c mice were shaved on the dorsal skin 1 day before modeling and randomly assigned to six groups: PBS, CGRP, LL37, CGRP + LL37, CGRP + LL37 + ritlecitinib, and CGRP + LL37 + rimegepant. Mice in the PBS and CGRP groups received intradermal injections of 40 μL PBS or CGRP solution, respectively. Mice in the LL37 group received intradermal injections of 40 μL LL37 peptide every 12 h for two consecutive days. Mice in the CGRP + LL37 group were pretreated with CGRP once 12 h before model establishment and then received intradermal injections of 40 μL of the mixed solution containing LL37 and CGRP.

For pharmacological intervention experiments, rimegepant and ritlecitinib were administered by oral gavage at doses of 20 mg/kg/day and 15 mg/kg/day, respectively, for 14 consecutive days. During the final 2 days of treatment, mice received intradermal injections of the CGRP and LL37 mixed solution as described above (28, 30). Skin lesion severity was evaluated according to redness score and redness area. Redness score was graded on a scale from 0 to 4, where 0 indicated no visible symptoms, 1 slight redness, 2 moderate redness, 3 severe redness, and 4 very severe redness. Redness area was quantified using ImageJ Fiji software (version 1.54g).

### 2.5 *In vivo* antibody-mediated mast-cell depletion

ACK2-mediated *in vivo* mast-cell depletion through blockade of the KIT pathway has been widely used and validated in multiple experimental disease models (34–36). Because ACK2-mediated mast-cell depletion has been reported to achieve efficient and reproducible depletion in C57BL/6 mice, this strain was selected for mast-cell depletion experiments (37–39). Eight-week-old female C57BL/6 mice were randomly divided into experimental groups, with five mice in each group. Mast-cell lineage depletion was induced by intraperitoneal administration of an InVivo anti-mouse c-Kit/CD117 antibody (clone ACK2; BioXCell, BE0293) at 500 μg per mouse every 2 days for 2 weeks. Control mice were treated with the corresponding isotype control antibody.

### 2.6 Flow cytometric analysis

Flow cytometric analysis was performed to confirm the depletion efficiency of bone marrow mast cell progenitors (MCps). According to previously reported immunophenotypic criteria, murine MCps were identified as CD117^+^ integrin β7^+^ cells within the CD45^+^SSC^low^ population, allowing enrichment of progenitor-like mast-cell populations while reducing contamination from mature mast cells (40).

Femurs and tibias were harvested from C57BL/6 mice, and bone marrow cells were flushed out and prepared as single-cell suspensions (41). Suspensions were filtered through a 40-μm cell strainer, and red blood cells were removed by incubation with ACK lysis buffer (Solarbio, R1013) at room temperature. Cells were washed and resuspended in FACS buffer containing 2% FBS and 1 mM EDTA in PBS at approximately 1 × 10^7^ cells/mL. For each sample, 100 μL of cell suspension was used for staining. Before staining, cells were incubated with anti-CD16/CD32 antibody (Invitrogen, 14-0161-82) to block Fc receptors and reduce nonspecific binding. Cells were then stained for 30 min at 4 °C in the dark with the following fluorochrome-conjugated antibodies: FITC-conjugated anti-mouse CD45 (Invitrogen, 11-0451-82), APC-conjugated anti-mouse CD117 (Invitrogen, 17-1171-82), and PE-conjugated anti-mouse integrin β7 (Invitrogen, 12-5867-42). All antibodies were used according to the manufacturers’ instructions. Data were acquired using a Mindray BriCyte MX flow cytometer and analyzed with FlowJo software (version 10.8.1.).

### 2.7 Histological analysis

Skin samples were fixed in 4% paraformaldehyde for 24 h, embedded in paraffin, and sectioned at a thickness of 4 μm. Hematoxylin and eosin (H&E) staining was performed according to the manufacturer’s instructions (Sangon Biotech, E607318). Mast cells were visualized using toluidine blue staining (Sangon Biotech, E670105). Microscopic evaluation was performed under a light microscope. For each specimen, three high-power fields were randomly selected from three sections to quantify dermal inflammatory infiltration and mast-cell density.

### 2.8 Datasets and bioinformatic analysis

The rosacea microarray dataset GSE65914 was downloaded from the Gene Expression Omnibus database. The GSE65914 dataset contains 58 samples. For the present analysis, 32 samples, including 12 papulopustular rosacea samples and 20 healthy control samples, were used as the training set (42). After standard preprocessing, differentially expressed genes (DEGs) between rosacea lesions and normal skin were identified using the “limma” package in R. The thresholds were set as |log_2_FC| ≥ 0.5 and adjusted *P* < 0.05. DEGs results were visualized using the “heatmap” and “volcano” packages.

Functional enrichment analyses, including Gene Ontology (GO) and Kyoto Encyclopedia of Genes and Genomes (KEGG) pathway analyses, were performed using “clusterProfiler”, “enrichplot”, and “ggplot2” (43). Gene set enrichment analysis (GSEA) was conducted using the “clusterProfiler” R package with the JAK–STAT signaling pathway gene set derived from MSigDB version 7.5. GSEA results and key driving genes within the pathway were visualized and annotated using “GseaVis” and “ggplot2” (44).

Immune-cell composition in GSE65914 was estimated using the CIBERSORT algorithm with the LM22 leukocyte signature matrix. CIBERSORT infers the relative proportions of 22 immune-cell subsets from bulk transcriptomic data through computational deconvolution (45). CIBERSORT-estimated immune-cell proportions were retained for downstream analysis. Spearman correlation analysis was used to evaluate the associations between the expression of CGRP receptor-related genes, including CALCRL and RAMP1, and the CIBERSORT-estimated abundance of resting mast cells. Correlations were visualized using scatter plots with fitted regression lines.

### 2.9 Cell counting kit-8 assay

Cell viability of LUVA mast cells was assessed using the Cell Counting Kit-8 (CCK-8) assay according to the manufacturer’s instructions. LUVA cells were seeded into 96-well plates at a density of 1 × 10^4^ cells per well and incubated under the indicated conditions. Subsequently, 10 μL of CCK-8 solution was added to each well, followed by incubation at 37 °C for 1 h. Optical density at 450 nm was measured using a microplate reader (Bio-Tek Instruments, MQX200, USA). Data were statistically analyzed and graphed using GraphPad Prism 10.0.

### 2.10 Cellular and tissue immunofluorescence analysis

Cellular immunofluorescence staining was performed to evaluate the expression and localization of the CGRP receptor subunits CALCRL and RAMP1 and the activation status of the JAK3–STAT1 pathway in LUVA cells. LUVA cells were seeded onto poly-L-lysine-coated coverslips (Biosharp, BS-14-RC) and allowed to adhere before staining. Coverslips bearing LUVA cells were fixed with 4% paraformaldehyde for 15 min at room temperature, washed three times with PBS, permeabilized with 0.2% Triton X-100 for 15 min, and blocked with 3% goat serum for 1 h.

Tissue immunofluorescence staining was performed to evaluate CGRP expression and to determine whether CALCRL, RAMP1, p-JAK3, and p-STAT1 signals were localized in CD117^+^ mast cells in lesional skin from patients with rosacea. Paraffin-embedded sections were deparaffinized in xylene, rehydrated through graded ethanol, and subjected to antigen retrieval with EDTA buffer at pH 8.0. After permeabilization with 0.2% Triton X-100 for 20 min, sections were blocked with 10% goat serum to reduce nonspecific binding.

For both cellular and tissue immunofluorescence staining, samples were incubated with primary antibodies overnight at 4 °C. After three washes with PBS, samples were incubated with Alexa Fluor 488-conjugated goat anti-rabbit IgG (H+L) (ABclonal, AS053) or Alexa Fluor 594-conjugated goat anti-mouse IgG (H+L) (ABclonal, AS054) for 1 h at room temperature in the dark. Nuclei were counterstained with DAPI (SparkJade, EE0011), and samples were mounted with antifade mounting medium.

The following primary antibodies were used: anti-CGRP (1:200, Cell Signaling Technology, 14959), anti-CALCRL (1:200, Alomone Labs, ACR-060), anti-RAMP1 (1:200, Invitrogen, PA5-110265), anti-phospho-JAK3 (1:100, Invitrogen, PA5-105892), anti-phospho-STAT1 (1:100, Cell Signaling Technology, 9167), and anti-CD117/c-Kit (1:200, Santa Cruz Biotechnology, sc-365504). Fluorescence images were captured using an Olympus FV3000 confocal microscope (Olympus, Japan). For each sample, three fields within the lesional dermis were randomly selected under the same magnification.

### 2.11 Molecular docking

The active human CGRP receptor complex structure (PDB ID: 6E3Y) and the crystal structure of the JAK3 kinase domain (PDB ID: 1YVJ) were retrieved from the Protein Data Bank. The CALCRL chain was extracted from the 6E3Y complex and prepared for docking. Protein–protein docking between CALCRL and JAK3 was performed using the H-DOCK server version 1.1 (46). The best-ranked docking pose was selected for subsequent interaction-interface analysis. The predicted CALCRL–JAK3 complex was submitted to the Protein–Ligand Interaction Profiler (PLIP) server for systematic identification of residue-level non-covalent interactions, with JAK3 set as the reference chain. Key interface residues and interaction types were further inspected and visualized using PyMOL version 2.4 (47).

### 2.12 Co-immunoprecipitation

After 24–48 h of transfection, HEK293T cells were washed twice with cold PBS and lysed on ice using IP lysis buffer (Thermo Fisher Scientific, 87787) supplemented with protease and phosphatase inhibitors. Cell lysates were centrifuged at 12,000 × g for 15 min at 4 °C, and the clarified supernatants were collected. Protein concentrations were determined using a BCA protein assay kit, and equal amounts of total protein were used for each immunoprecipitation reaction. A small aliquot of each lysate was retained as the input control. For reciprocal co-immunoprecipitation, the remaining lysates were divided into two portions and incubated with either 20 μL of anti-FLAG magnetic beads (Selleck, B26102) or 20 μL of Pierce anti-HA magnetic beads (Thermo Fisher Scientific, 88836) overnight at 4 °C with gentle rotation. The next day, beads were washed four times with PBST containing 0.1% Tween-20 in PBS at pH 7.4. Input and immunoprecipitated proteins were analyzed by western blotting using anti-DYKDDDDK tag antibody (1:1000, Cell Signaling Technology, 14793) and anti-HA tag antibody (1:8000, Proteintech Group, 51064-2-AP), followed by HRP-linked anti-rabbit IgG secondary antibody (Proteintech Group, SA00001-2).

### 2.13 Western blotting

Total protein was extracted using RIPA lysis buffer and quantified using a BCA assay kit (SparkJade, EC0001-A). Equal amounts of protein were separated on 8%–12% SDS-PAGE gels and transferred onto PVDF membranes (Millipore, USA). Membranes were blocked with 5% skim milk for 1 h and incubated with primary antibodies overnight at 4 °C. After three washes with TBST, membranes were incubated with HRP-linked anti-rabbit IgG secondary antibody (Proteintech Group, SA00001-2) for 1 h at room temperature. Protein bands were visualized using enhanced chemiluminescence reagent (ZEN-BIOscience, 17046) and imaged using the SH-Magic 523Mini multifunctional chemiluminescence imaging system (Shenhua Technology, China). Grayscale values were quantified using ImageJ software version 1.46r.

The following primary antibodies were used: anti-CALCRL (1:500, Alomone Labs, ACR-060), anti-RAMP1 (1:1000, ZEN-BIOscience, R25544), anti-JAK3 (1:1000, Cell Signaling Technology, 8827), anti-STAT1 (1:1000, ZEN-BIOscience, R25799), anti-phospho-JAK3 (1:750, Cell Signaling Technology, 5031), anti-phospho-STAT1 (1:500, Cell Signaling Technology, 9167), anti-PKA (1:1000, ZEN-BIOscience, 864496), anti-phospho-PKA (1:1000, ZEN-BIOscience, 347334), anti-α-tubulin (1:6000, Proteintech, 11224-1-AP), anti-β-actin (1:5000, Proteintech, 20536-1-AP), and anti-GAPDH (1:3000, Cell Signaling Technology, 2118).

### 2.14 Reverse transcription and quantitative real-time PCR

Total RNA was isolated using Sparkzol reagent (SparkJade, AC0101-B). RNA samples of 1 μg were reverse transcribed into cDNA using MightyScript First Strand cDNA Synthesis Master Mix (Sangon Biotech, B639251). Quantitative real-time PCR was performed using SGExcel FastSYBR Mixture (Sangon Biotech, B532955) on a Quant Gene 9600 Real-Time PCR System. Gene expression was normalized to GAPDH, and relative expression levels were calculated using the 2^–ΔΔCt^ method. The primer sequences were as follows: CALCRL forward, 5′-ACTCTTTCCCACCTTGCTTGT-3′ and reverse, 5′-AGGGGTCAAGACCCAGTCC-3′; RAMP1 forward, 5′-GGAGGCTAACTACGGTGCC-3′ and reverse, 5′-CTCCCTGTAGCTCCTGATGG-3′; GAPDH forward, 5′-GGGCTCTCCAGAACATCATC-3′ and reverse, 5′-TTCTAGACGGCAGGTCAGGT-3′.

### 2.15 Degranulation assay

LUVA cell degranulation was assessed by quantifying extracellular β-hexosaminidase release (48). LUVA cells were treated according to the indicated experimental conditions, and the negative control group received an equal volume of vehicle. After incubation at 37 °C, cells were centrifuged, and supernatants and cell pellets were collected separately. Cell pellets were lysed with 0.5% Triton X-100. Equal volumes of supernatants and cell lysates were incubated with p-nitrophenyl-N-acetyl-β-D-glucosaminide substrate at 37 °C for 90 min, followed by the addition of 0.2 M glycine-NaOH buffer at pH 10.7 to terminate the reaction. The β-hexosaminidase activity was measured at 405 nm using a microplate reader. The percentage of β-hexosaminidase release was calculated using the following formula:


$$\text{β‐hexosaminidase release }(\%)=[{\text{OD}}_{405}\text{ of supernatant}/({\text{OD}}_{405}\text{ of supernatant}+{\text{OD}}_{405}\text{ of cell lysate})]\times 100$$

### 2.16 Enzyme-linked immunosorbent assay

Serum samples from patients with rosacea and healthy controls were centrifuged, and CGRP concentrations were measured using a CGRP ELISA kit (Jianglaibio, JL11472). For LUVA cell experiments, culture supernatants were collected after 24 h of treatment for inflammatory mediator detection. For histamine measurement, supernatants were collected after 30 min of stimulation. Dorsal skin tissues from the modeling and administration sites of mice in each group were harvested and homogenized. Histamine levels were measured using a histamine ELISA kit (Jianglaibio, JL45802). Inflammatory mediator levels, including IL-6, TNF-α, CCL2, CXCL10, and VEGF, in cell culture supernatants and mouse skin tissue homogenates were measured using corresponding ELISA kits (Jianglaibio, Shanghai, China). β-hexosaminidase levels in mouse skin tissue homogenates were measured using a β-hexosaminidase ELISA kit (Jianglaibio, JL20214). All assays were performed according to the manufacturers’ instructions.

Intracellular cAMP levels in LUVA mast cells were quantified using a commercial ELISA kit (Elabscience, E-EL-0056c). LUVA cells were pretreated with FSK (10 μM) or suramin (100 μM) for 10 min and then stimulated with CGRP for 10 min in the presence of FSK or suramin. After treatment, cells were collected and transferred to 1.5 mL Eppendorf tubes. Cells were disrupted by sonication for 3-s with 3-s intervals, repeated three times. After disruption, samples were centrifuged at 12,000 rpm for 10 min at 4 °C, and the supernatants were collected for analysis. cAMP content was determined according to the manufacturer’s protocol. Optical density was measured using a microplate reader (Thermo Scientific, USA), and concentrations were calculated from standard curves.

### 2.17 Statistical analysis

Data are presented as the mean ± standard deviation unless otherwise indicated. Statistical analyses and graph generation were performed using GraphPad Prism 10.0. Comparisons between two groups were performed using unpaired two-tailed Student’s *t*-tests. Welch’s correction was applied when variances were unequal. Comparisons among multiple groups were performed using one-way or two-way analysis of variance, as appropriate, followed by the indicated *post hoc* multiple-comparison tests. Spearman’s correlation coefficient was used for correlation analyses. A *P* value < 0.05 was considered statistically significant.

## 3 Results

### 3.1 CGRP receptor gene upregulation is associated with JAK–STAT signaling and altered mast-cell status in rosacea lesions

**Figure 1:**
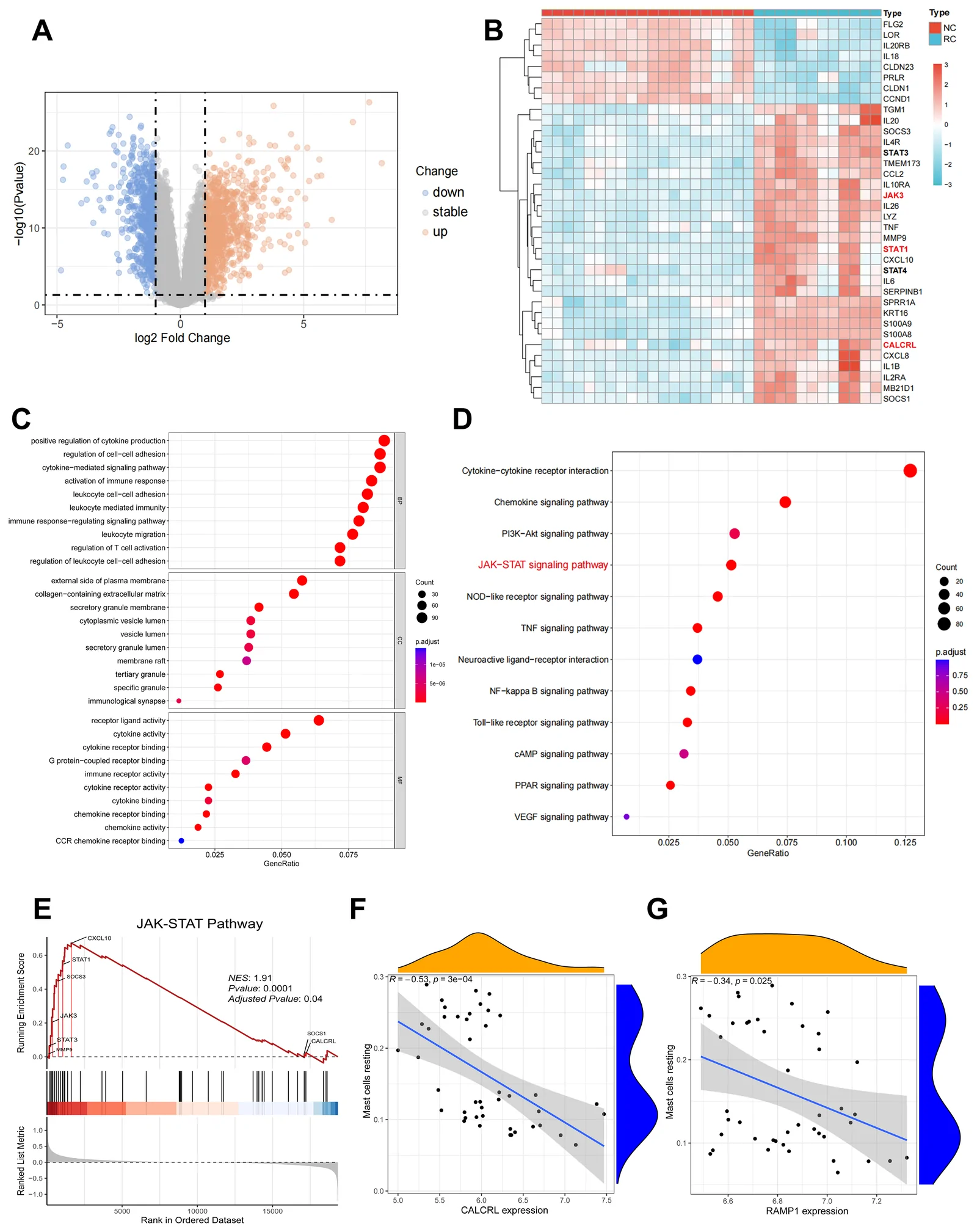
CGRP receptor genes are upregulated and associated with JAK–STAT pathway activation and altered mast-cell status in rosacea lesions. **(A, B)** Volcano plot **(A)** and heatmap **(B)** showing differentially expressed genes (DEGs) between papulopustular rosacea lesions and healthy control skin. The threshold was set as adjusted *P* < 0.05 and |log_2_FC| ≥ 0.5. **(C)** Dot plot showing Gene Ontology enrichment analysis of biological processes, cellular components, and molecular functions among DEGs. **(D)** Dot plot showing KEGG pathway enrichment analysis of DEGs, with inflammatory and immune-related pathways, including the JAK–STAT signaling pathway, highlighted. **(E)** Gene set enrichment analysis (GSEA) of the GSE65914 dataset showing enrichment of the JAK–STAT signaling pathway in rosacea lesions. NES, normalized enrichment score. An adjusted *P* value < 0.05 was considered statistically significant. **(F, G)** Scatter plots showing correlations between CALCRL **(F)** or RAMP1 **(G)** expression and the CIBERSORT-estimated proportion of resting mast cells in papulopustular rosacea lesions.

To explore the molecular mechanisms underlying rosacea, we analyzed the human skin transcriptomic dataset GSE65914. Differential expression analysis identified 925 upregulated and 683 downregulated genes in papulopustular rosacea lesions (PPR) compared with healthy skin, as visualized by volcano plot analysis (**Figure 1A**). Hierarchical clustering further showed increased relative expression of selected JAK–STAT signaling-related genes, together with the core CGRP receptor component CALCRL, in rosacea lesions compared with healthy control skin (**Figure 1B**).

Gene Ontology (GO) enrichment analysis revealed prominent dysregulation of biological processes and cellular components related to immune activation, inflammatory mediator production, and secretory granule function among the differentially expressed genes (**Figure 1C**). Consistently, Kyoto Encyclopedia of Genes and Genomes (KEGG) pathway enrichment analysis showed that these genes were enriched in inflammatory and immune-related pathways, including cytokine–cytokine receptor interaction, chemokine signaling, and JAK–STAT signaling. Notably, the enrichment of the neuroactive ligand–receptor interaction pathway suggested that neuroimmune signaling may also contribute to the transcriptional changes observed in rosacea lesions (**Figure 1D**). Gene set enrichment analysis further confirmed significant enrichment of the JAK–STAT signaling pathway in rosacea lesions, with a normalized enrichment score (NES) of 1.91 and an adjusted *P* value of 0.004. CALCRL was also highlighted within the ranked gene list, supporting a potential link between CGRP receptor-related signaling and JAK–STAT pathway activation in rosacea skin (**Figure 1E**). In addition, correlation analysis showed that CALCRL and RAMP1 expression were negatively correlated with the CIBERSORT-estimated proportion of resting mast cells (**Figures 1F, G**). This inverse association may reflect a shift in mast-cell status within rosacea lesions, consistent with the possibility that increased CGRP receptor-related signaling is associated with reduced resting mast-cell signatures and enhanced mast-cell activation.

### 3.2 Serum CGRP is elevated in rosacea and correlates with flushing and erythema severity

**Figure 2:**
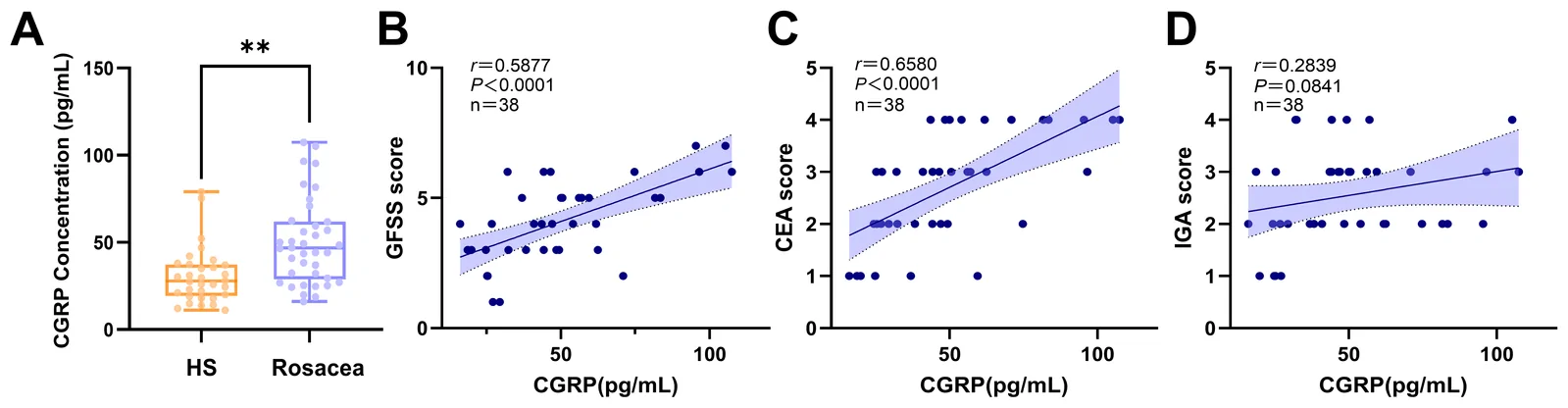
Serum CGRP is elevated in rosacea and correlates with flushing and erythema severity. **(A)** Serum CGRP concentrations in healthy subjects (HS; *n* = 32) and patients with rosacea (*n* = 38) were measured by ELISA. Box plots show the median, interquartile range, and minimum-to-maximum values. Statistical significance was determined using a two-tailed unpaired *t*-test with Welch’s correction. **P < 0.01. **(B–D)** Correlation analyses between serum CGRP concentration and Global Flushing Severity Score (GFSS), Clinician’s Erythema Assessment (CEA), and Investigator’s Global Assessment (IGA) scores in patients with rosacea (*n* = 38). Spearman’s correlation coefficient was used for correlation analysis.

Previous studies have reported increased circulating CGRP levels in patients with rosacea, suggesting a potential role for CGRP in rosacea-associated neurovascular inflammation. To validate this observation in our clinical cohort and determine its relationship with disease-related manifestations, we measured serum CGRP levels in patients with rosacea and healthy controls. Serum CGRP concentrations were significantly higher in patients with rosacea than in healthy controls (55.24 ± 24.60 vs. 30.67 ± 15.97 pg/mL; **Figure 2A**).

We next examined the association between serum CGRP levels and clinical severity scores, including the Global Flushing Severity Score (GFSS), Clinician’s Erythema Assessment (CEA), and Investigator’s Global Assessment (IGA). Serum CGRP levels were positively correlated with GFSS scores (*r* = 0.5877, *P* < 0.0001) and CEA scores (*r* = 0.6580, *P* < 0.0001), but not with the IGA scores (*r* = 0.2839, *P* = 0.0841; **Figures 2B–D**). These findings suggest that increased serum CGRP levels are more closely associated with flushing and erythema than with overall lesion severity, supporting a potential role for CGRP in rosacea-related neurovascular responses.

### 3.3 Increased mast-cell infiltration and mast cell-associated CGRP receptor–JAK3–STAT1 signaling in rosacea lesions

**Figure 3:**
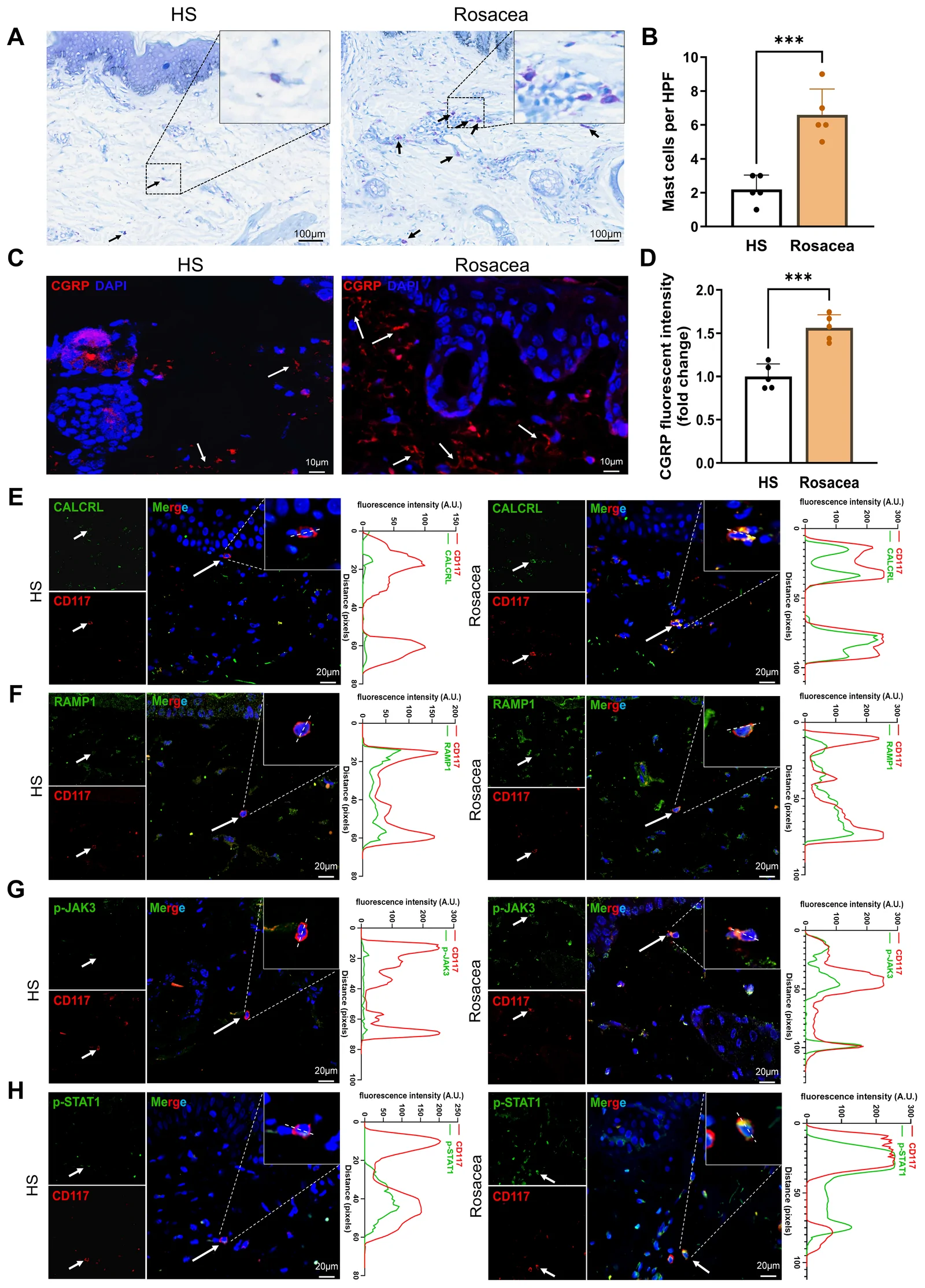
Increased mast-cell infiltration and mast cell-associated CGRP receptor–JAK3–STAT1 signaling in rosacea lesions. **(A)** Representative toluidine blue staining of skin sections from healthy subjects (HS) and patients with rosacea. Black arrows indicate dermal mast cells. Scale bars, 100 μm. **(B)** Quantification of dermal mast-cell numbers per high-power field (HPF) in HS and rosacea skin samples (*n* = 5 per group). **(C)** Representative immunofluorescence images showing CGRP expression in skin sections from HS and patients with rosacea. White arrows indicate CGRP-positive signals in the dermis. Scale bars, 10 μm. **(D)** Quantification of relative dermal CGRP fluorescence intensity (*n* = 5 per group). **(E–H)** Representative confocal images and line-scan analyses showing overlapping signals of CALCRL **(E)**, RAMP1 **(F)**, p-JAK3 **(G)**, or p-STAT1 **(H)** with CD117^+^ mast cells in the dermis of HS and rosacea skin. Scale bars, 20 μm. Data are presented as mean ± SD. Statistical significance was determined using a two-tailed unpaired *t*-test with Welch’s correction. ***P < 0.001.

To validate the transcriptomic findings at the tissue level and determine their cellular localization in rosacea lesions, we performed histological and immunofluorescence analyses using skin samples from patients with rosacea and healthy controls. Toluidine blue staining showed a significant increase in dermal mast-cell density in rosacea lesions compared with healthy skin (**Figures 3A, B**). Consistent with the elevated serum CGRP levels described above, immunofluorescence staining revealed increased CGRP immunoreactivity in rosacea lesions (**Figures 3C, D**).

We next examined the localization of the canonical CGRP receptor components CALCRL and RAMP1. Both CALCRL and RAMP1 signals were detected in CD117^+^ mast cells in rosacea lesions, indicating that lesional mast cells may be responsive to CGRP signaling (**Figures 3E, F**). Given the enrichment of JAK–STAT signaling in transcriptomic analysis, we further assessed the activation status of this pathway in lesional mast cells. Increased p-JAK3 and p-STAT1 signals were observed in rosacea lesions and showed marked colocalization with CD117^+^ mast cells (**Figures 3G, H**; **Supplementary Figures 1A–D**). Together, these findings support the tissue-level activation of a mast cell-associated CGRP receptor–JAK3–STAT1 signaling axis in rosacea lesions.

### 3.4 CGRP activates CALCRL/RAMP1–JAK3–STAT1 signaling and promotes mast-cell degranulation

Based on the lesion-associated activation of the CGRP receptor–JAK3–STAT1 axis, we next investigated whether CGRP directly activates mast cells and explored the underlying intracellular signaling mechanism using LUVA mast cells. We first assessed the effect of CGRP on LUVA cell viability. CCK-8 assays showed that CGRP treatment at concentrations ranging from 0 to 200 nM did not significantly impair LUVA cell viability over 0–72 h (**Supplementary Figure 2A**). In parallel, CGRP stimulation for 30 min induced a marked increase in β-hexosaminidase release, with 100 nM CGRP producing a robust degranulation response without detectable cytotoxicity (**Supplementary Figure 2B**). Therefore, 100 nM CGRP was selected for subsequent *in vitro* stimulation experiments.

**Figure 4:**
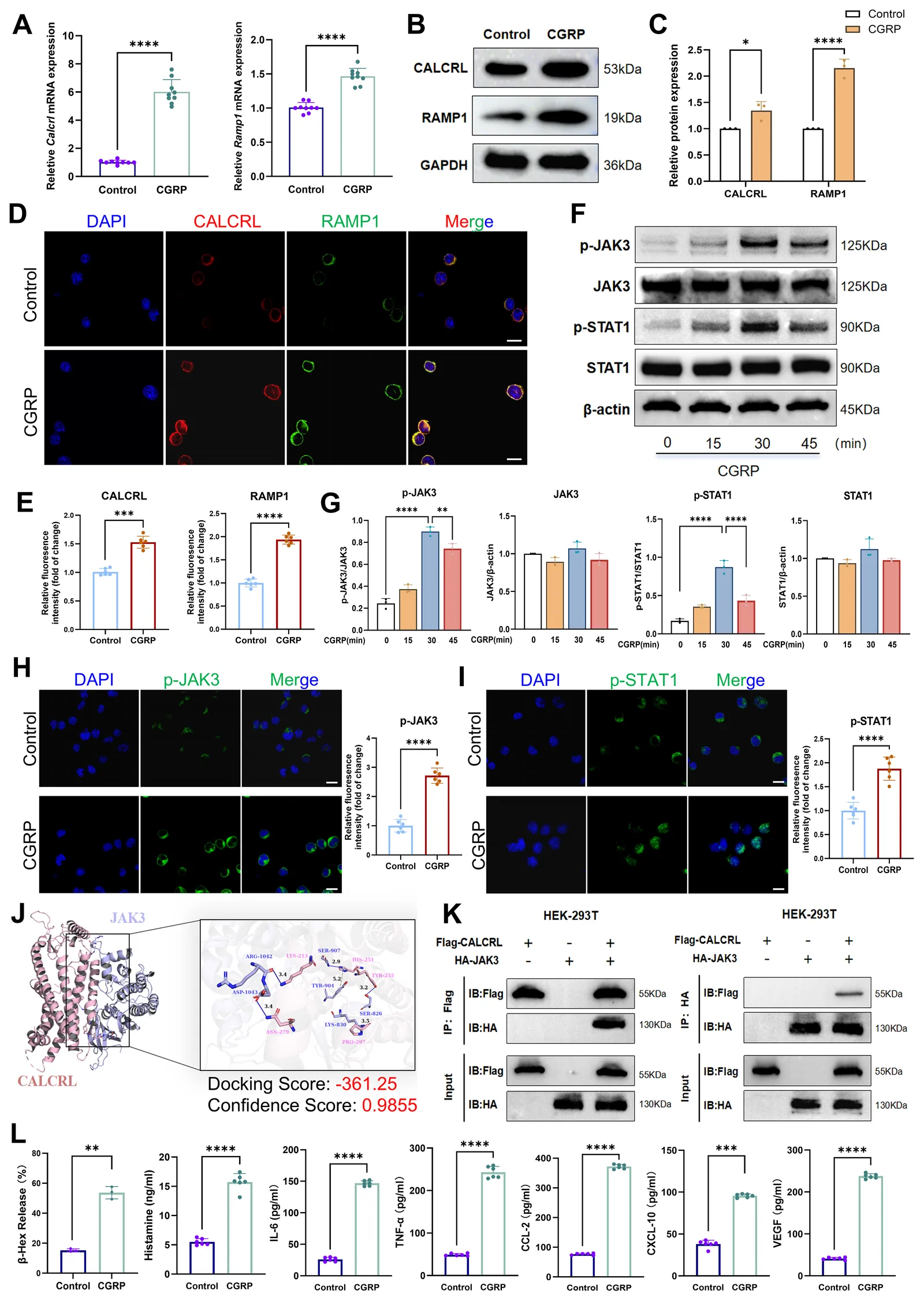
CGRP activates CALCRL/RAMP1–JAK3–STAT1 signaling and promotes mast-cell degranulation. **(A)** RT-qPCR analysis of CALCRL and RAMP1 mRNA levels in control and CGRP-treated LUVA cells. LUVA cells were treated with CGRP at 100 nM for 24 h (*n* = 9 per group). **(B, C)** Western blotting analysis **(B)** and quantification **(C)** of CALCRL and RAMP1 protein levels in LUVA cells treated with CGRP at 100 nM for 48 h. **(D, E)** Representative confocal immunofluorescence images **(D)** and quantification **(E)** showing CALCRL and RAMP1 expression in LUVA cells after CGRP stimulation. DAPI was used for nuclear staining. Scale bars, 20 μm. **(F, G)** Western blotting analysis **(F)** and quantification **(G)** of p-JAK3, JAK3, p-STAT1, and STAT1 levels in LUVA cells stimulated with CGRP at 100 nM for 0, 15, 30, and 45 min. **(H, I)** Representative confocal immunofluorescence images and quantification showing p-JAK3 **(H)** and p-STAT1 **(I)** signals in LUVA cells after CGRP stimulation. DAPI was used for nuclear staining. Scale bars, 20 μm. **(J)** Molecular docking model of CALCRL and JAK3. CALCRL is shown in pink and JAK3 in purple. The boxed panel shows an enlarged view of the predicted interaction interface. **(K)** Reciprocal co-immunoprecipitation analysis showing the association between Flag-CALCRL and HA-JAK3 in HEK293T cells. **(L)** β-hexosaminidase release rate (*n* = 3 per group) and ELISA analysis of histamine release and inflammatory mediator secretion, including IL-6, TNF-α, CCL2, CXCL10, and VEGF (*n* = 6 per group), from LUVA cells after CGRP stimulation. Western blotting experiments were independently repeated three times. Data are presented as mean ± SD. Statistical significance was determined using an unpaired two-tailed Student’s *t*-test for comparisons between two groups and one-way ANOVA followed by Tukey’s multiple-comparisons test for comparisons among multiple groups. ns, not significant; *P < 0.05, **P < 0.01, ***P < 0.001, ****P < 0.0001.

We then examined whether CGRP regulates the expression of its receptor components in LUVA mast cells. CGRP stimulation increased CALCRL and RAMP1 expression at both the mRNA and protein levels (**Figures 4A–C**). Consistently, confocal immunofluorescence showed enhanced CALCRL and RAMP1 signals in CGRP-treated LUVA cells (**Figures 4D, E**).

Next, we assessed JAK3–STAT1 pathway activation following CGRP stimulation. Western blotting analysis revealed rapid phosphorylation of JAK3 and STAT1 after CGRP exposure, with increased p-JAK3 and p-STAT1 levels observed within 15–45 min, whereas total JAK3 and STAT1 levels remained largely unchanged (**Figures 4F, G**). Confocal microscopy further confirmed increased p-JAK3 and p-STAT1 signals in CGRP-treated LUVA cells (**Figures 4H, I**).

To explore a potential molecular link between CGRP receptor activation and JAK3–STAT1 signaling, we performed protein–protein docking analysis between CALCRL and JAK3. Docking analysis predicted a favorable CALCRL–JAK3 interaction model, with a docking score of −361.25 and a confidence score of 0.9855. The predicted interface was supported by multiple hydrogen bonds and a π–π stacking interaction (**Figure 4J**). To further examine this potential association experimentally, Flag-CALCRL and HA-JAK3 were co-transfected into HEK293T cells, followed by reciprocal co-immunoprecipitation. Co-immunoprecipitation analysis detected Flag-CALCRL and HA-JAK3 in the corresponding immunoprecipitates, supporting a physical association between CALCRL and JAK3 in this overexpression system (**Figure 4K**).

Finally, we evaluated the functional consequences of CGRP stimulation in LUVA mast cells. CGRP markedly increased β-hexosaminidase and histamine release, as well as the secretion of IL-6, TNF-α, CCL2, CXCL10, and VEGF (**Figure 4L**), indicating that CGRP promotes a pro-inflammatory mast-cell response. Together, these findings suggest that CGRP activates LUVA mast cells and engages CALCRL/RAMP1-associated JAK3–STAT1 signaling, thereby promoting mast-cell degranulation and inflammatory mediator release.

### 3.5 CGRP-induced mast-cell activation is attenuated by blockade of the CGRP receptor–JAK3–STAT1 axis

**Figure 5:**
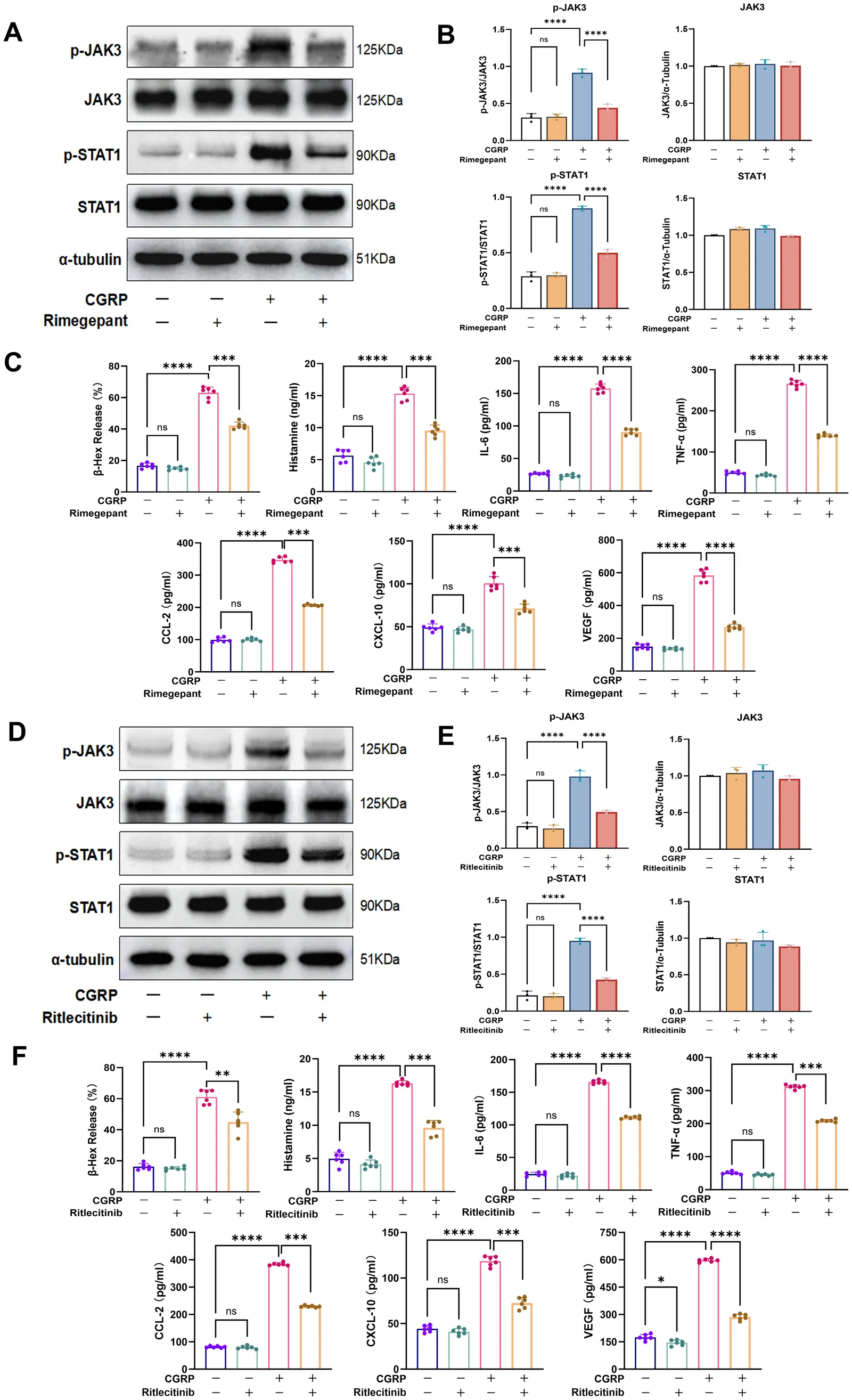
CGRP-induced mast-cell activation is attenuated by blockade of the CGRP receptor–JAK3–STAT1 axis. **(A, B)** Western blotting analysis **(A)** and quantification **(B)** of p-JAK3, JAK3, p-STAT1, and STAT1 levels in LUVA cells treated with rimegepant and/or CGRP. Cells were preincubated with or without rimegepant at 1 μM for 30 min and then stimulated with or without CGRP before collection. **(C)** β-hexosaminidase release rate and ELISA quantification of histamine release, IL-6, TNF-α, CCL2, CXCL10, and VEGF secretion in LUVA cells following rimegepant and/or CGRP treatment (*n* = 6 per group). **(D, E)** Western blotting analysis **(D)** and quantification **(E)** of p-JAK3, JAK3, p-STAT1, and STAT1 levels in LUVA cells treated with ritlecitinib and/or CGRP. Cells were preincubated with or without ritlecitinib at 500 nM for 30 min and then stimulated with or without CGRP before collection. **(F)** β-hexosaminidase release rate and ELISA analysis of histamine release, IL-6, TNF-α, CCL2, CXCL10, and VEGF secretion in LUVA cells following ritlecitinib and/or CGRP treatment (*n* = 6 per group). Western blotting experiments were independently repeated three times. Data are presented as mean ± SD. Statistical significance was determined using one-way ANOVA followed by Tukey’s multiple-comparisons test. ***P < 0.001, ****P < 0.0001.

To determine whether CGRP promotes mast-cell activation through the CGRP receptor–JAK3–STAT1 signaling axis, LUVA cells were pretreated with the CGRP receptor antagonist rimegepant or the selective JAK3 inhibitor ritlecitinib before CGRP stimulation. Western blotting analysis showed that rimegepant markedly reduced CGRP-induced phosphorylation of JAK3 and STAT1, without obvious changes in total JAK3 or STAT1 expression (**Figures 5A, B**). Consistently, rimegepant significantly suppressed CGRP-induced β-hexosaminidase and histamine release and reduced the levels of IL-6, TNF-α, CCL2, CXCL10, and VEGF in culture supernatants (**Figure 5C**). Similarly, ritlecitinib pretreatment attenuated CGRP-induced JAK3 and STAT1 phosphorylation while leaving total JAK3 and STAT1 levels largely unchanged (**Figures 5D, E**). Ritlecitinib also decreased β-hexosaminidase and histamine release and reduced the production of IL-6, TNF-α, CCL2, CXCL10, and VEGF following CGRP stimulation (**Figure 5F**). These findings indicate that activation of the CGRP receptor–JAK3–STAT1 axis contributes to CGRP-induced mast-cell degranulation and inflammatory mediator release.

Because CGRP is known to activate the classical Gαs–cAMP–PKA pathway (26, 49), we next examined whether CGRP-induced JAK3–STAT1 activation was primarily mediated by this canonical signaling cascade. ELISA analysis showed that CGRP stimulation significantly increased intracellular cAMP levels in LUVA cells (**Supplementary Figure 3A**). Consistently, western blotting analysis revealed a time-dependent increase in PKA phosphorylation after CGRP stimulation, indicating activation of the cAMP–PKA pathway in LUVA cells (**Supplementary Figures 3B, C**).

To further assess the relationship between cAMP–PKA signaling and JAK3–STAT1 activation, we performed pharmacological intervention experiments using suramin, a G-protein inhibitor, and forskolin, an adenylyl cyclase activator. CCK-8 assays showed that suramin and forskolin did not exert significant cytotoxicity at 100 μM and 10 μM, respectively; these concentrations were therefore selected for subsequent experiments (**Supplementary Figures 4A, E**). ELISA analysis showed that suramin significantly reduced CGRP-induced cAMP accumulation (**Supplementary Figure 4B**). Western blotting analysis further confirmed that suramin inhibited CGRP-induced PKA phosphorylation but did not significantly alter CGRP-induced phosphorylation of JAK3 or STAT1 (**Supplementary Figures 4C, D**). Conversely, forskolin markedly increased intracellular cAMP levels in the absence or presence of CGRP stimulation (**Supplementary Figure 4F**). Western blotting analysis confirmed that forskolin enhanced PKA phosphorylation; however, it did not independently induce JAK3 or STAT1 phosphorylation and did not significantly affect CGRP-induced JAK3 or STAT1 phosphorylation (**Supplementary Figures 4G, H**). To further define the functional contribution of canonical G protein–cAMP–PKA signaling and STAT1-related signaling to CGRP-triggered mast-cell degranulation, we conducted dual-pathway inhibition assays in CGRP-stimulated LUVA cells using suramin and fludarabine. Suramin and fludarabine each markedly suppressed CGRP-elicited β-hexosaminidase and histamine release. Moreover, combined inhibitor treatment further decreased both degranulation markers relative to single-agent treatment (**Supplementary Figures 4I–K**). Collectively, these results indicate that CGRP-induced JAK3–STAT1 activation in LUVA mast cells is not primarily dependent on the canonical cAMP–PKA pathway. Functionally, however, both G protein–cAMP–PKA signaling and JAK3–STAT1 signaling contribute to CGRP-induced mast-cell degranulation.

### 3.6 Mast cells are required for CGRP-driven exacerbation of rosacea-like inflammation

**Figure 6:**
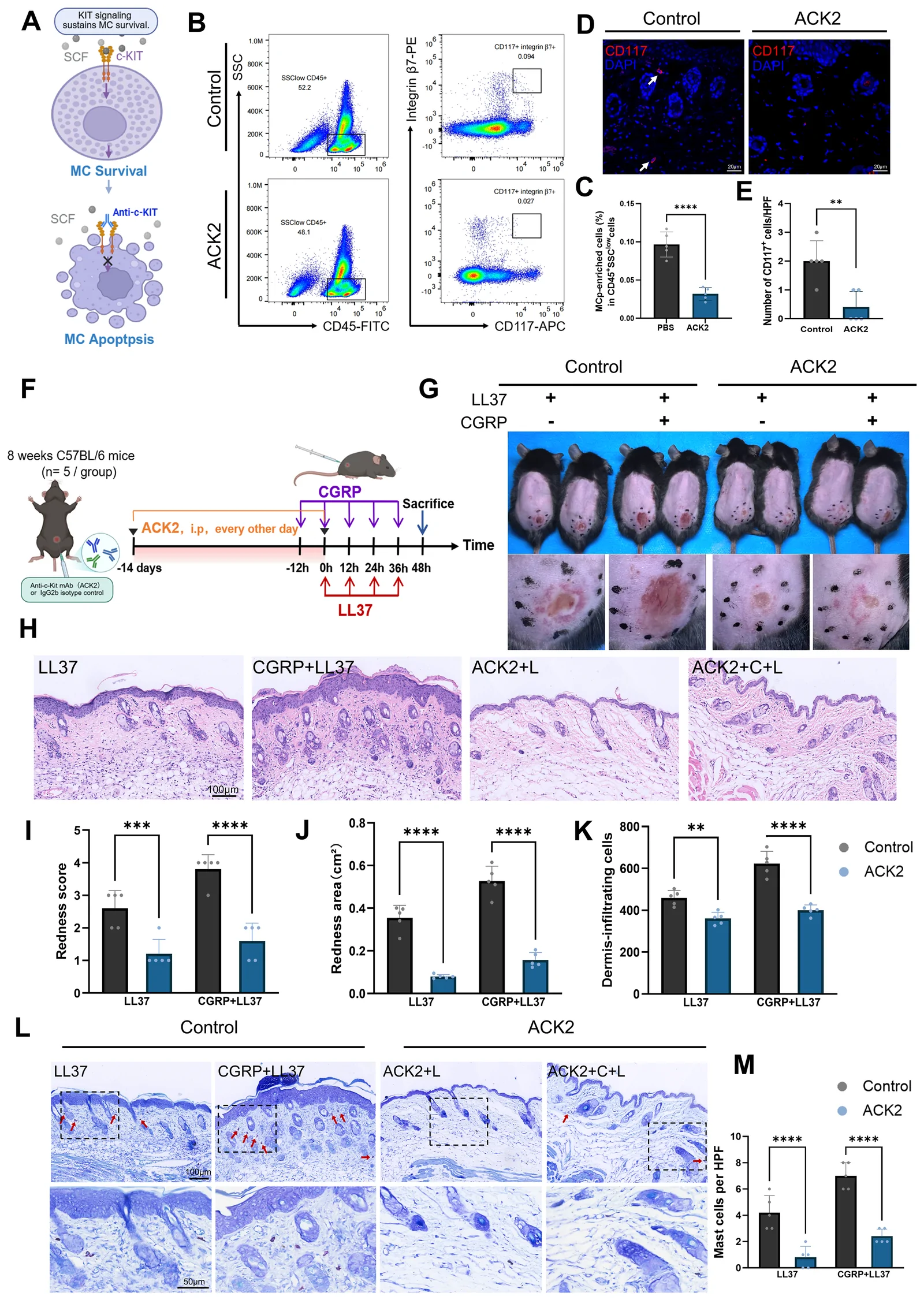
Mast cells are required for CGRP-driven exacerbation of rosacea-like inflammation. **(A)** Schematic illustration of ACK2-mediated blockade of c-Kit signaling and mast-cell lineage depletion. **(B, C)** Representative flow-cytometry plots **(B)** and quantitative analysis **(C)** of CD45^+^SSC^low^CD117^+^integrin β7^+^ mast cell progenitor-enriched cells in the bone marrow of control and ACK2-treated mice. **(D, E)** Representative immunofluorescence images **(D)** and quantification **(E)** of CD117^+^ cells in the dermis of control and ACK2-treated mice. White arrows indicate dermal CD117^+^ cells. Scale bars, 20 μm. **(F)** Schematic diagram of ACK2-mediated mast-cell depletion and CGRP administration in the LL37-induced rosacea-like mouse model. **(G, H)** Representative photographs of dorsal skin lesions **(G)** and corresponding H&E-stained skin sections **(H)** in each group. Scale bar, 100 μm. **(I–K)** Quantification of redness score **(I)**, redness area **(J)**, and dermal inflammatory cell infiltration **(K)**. **(L, M)** Representative toluidine blue staining images **(L)** and quantification **(M)** of dermal mast cells in each group. Red arrows indicate dermal mast cells. Scale bars, 100 μm and 50 μm. Control refers to mice treated with the corresponding isotype control antibody. MCp, mast cell progenitor; L, LL37; C+L, CGRP + LL37; A+L, ACK2 + LL37; A+C+L, ACK2 + CGRP + LL37. *n* = 5 mice per group. Data are presented as mean ± SD. Statistical significance was determined using a two-tailed unpaired *t*-test for two-group comparisons and two-way ANOVA followed by Šídák’s multiple-comparisons test for multiple-group comparisons. ns, not significant; **P < 0.01, ***P < 0.001, ****P < 0.0001.

Mast-cell survival depends on SCF–c-Kit signaling, and blockade of c-Kit/CD117 using the anti-mouse c-Kit/CD117 antibody (ACK2) can disrupt this survival pathway and induce mast-cell lineage depletion. Therefore, ACK2-mediated c-Kit/CD117 blockade was used to assess the contribution of mast cells to CGRP-driven exacerbation of rosacea-like inflammation *in vivo* (**Figure 6A**).

To monitor the efficiency of mast-cell lineage depletion, we evaluated both bone marrow mast cell progenitor-enriched cells and cutaneous mast cells (50). Flow-cytometric analysis of bone marrow cells showed that, within the CD45^+^SSC^low^ population, ACK2 treatment markedly reduced the proportion of CD117^+^integrin β7^+^ mast cell progenitor-enriched cells compared with the control group (0.094% vs. 0.027%; **Figures 6B, C**; **Supplementary Figure 5A**). Consistently, immunofluorescence staining of dorsal skin sections showed a substantial reduction in cutaneous CD117^+^ cells after ACK2 administration (**Figures 6D, E**), and toluidine blue staining further confirmed reduced dermal mast cells (**Supplementary Figure 5B**), supporting the successful establishment of a mast cell-depleted mouse model.

After ACK2-mediated depletion, mice were challenged with LL37 with or without CGRP according to the schedule shown in **Figure 6F**. Representative dorsal skin images showed that CGRP aggravated LL37-induced erythema in control mice, whereas this effect was visibly reduced in ACK2-treated mice (**Figure 6G**). Quantitative analysis further showed that CGRP significantly increased redness score and redness area in LL37-treated control mice, whereas these increases were markedly attenuated after ACK2 treatment (**Figures 6I, J**). H&E staining showed that CGRP enhanced LL37-induced dermal inflammatory infiltration in control mice, whereas this effect was markedly blunted after ACK2-mediated mast-cell depletion (**Figures 6H, K**). Toluidine blue staining further showed increased mast-cell accumulation in CGRP + LL37-treated control skin compared with LL37-treated skin, whereas ACK2 treatment markedly reduced mast-cell numbers in both LL37 and CGRP + LL37 groups (**Figures 6L, M**). Together, these findings indicate that mast-cell lineage depletion markedly limits CGRP-mediated amplification of LL37-induced rosacea-like inflammation.

### 3.7 Targeting the CGRP receptor–JAK3–STAT1 axis alleviates CGRP-aggravated rosacea-like dermatitis in mice

**Figure 7:**
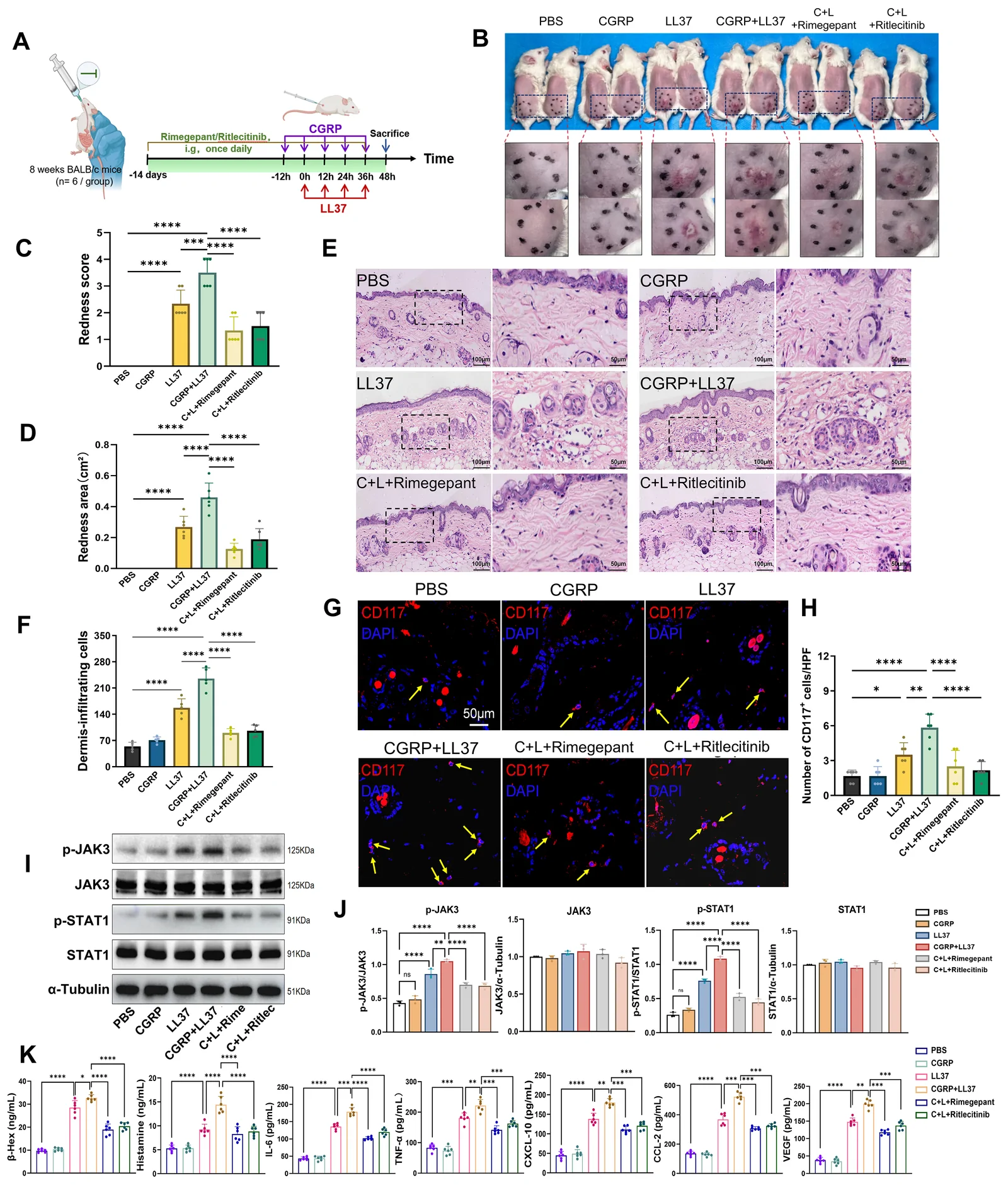
Targeting the CGRP receptor–JAK3–STAT1 axis alleviates CGRP-aggravated rosacea-like dermatitis in mice. **(A)** Schematic diagram of modeling and drug administration in the CGRP-aggravated LL37-induced rosacea-like dermatitis model. **(B–D)** Representative photographs of dorsal skin lesions **(B)** and quantification of redness score **(C)** and redness area **(D)** in each group. **(E, F)** Representative H&E staining images **(E)** and quantification **(F)** of dermal inflammatory cell infiltration. Scale bars, 100 μm and 50 μm. **(G, H)** Representative immunofluorescence images **(G)** and quantification **(H)** of CD117^+^ mast cells in the dermis. Scale bars, 50 μm. **(I, J)** Western blotting analysis **(I)** and quantification **(J)** of p-JAK3, JAK3, p-STAT1, and STAT1 levels in skin tissues. **(K)** ELISA analysis of β-hexosaminidase, histamine, IL-6, TNF-α, CXCL10, CCL2, and VEGF levels in skin tissues. *n* = 6 mice per group. Data are presented as mean ± SD. Statistical significance was determined using one-way ANOVA followed by Tukey’s multiple-comparisons test. ns, not significant; *P < 0.05, **P < 0.01, ***P < 0.001, ****P < 0.0001. C+L, CGRP + LL37.

We next evaluated whether pharmacological targeting of the CGRP receptor–JAK3–STAT1 axis could alleviate CGRP-aggravated rosacea-like inflammation *in vivo*. Mice were pretreated with rimegepant, a CGRP receptor antagonist, or ritlecitinib, a JAK3 inhibitor, and then subjected to LL37-induced rosacea-like dermatitis with CGRP administration (**Figure 7A**).

Representative dorsal skin images showed that CGRP exacerbated LL37-induced erythema, whereas rimegepant and ritlecitinib markedly alleviated this effect (**Figure 7B**). Consistently, both treatments significantly decreased redness score and redness area in CGRP + LL37-treated mice (**Figures 7C, D**). Histological analysis demonstrated that CGRP enhanced LL37-induced dermal inflammatory cell infiltration, whereas rimegepant and ritlecitinib markedly attenuated histological inflammation (**Figures 7E, F**). Immunofluorescence staining revealed an increased number of dermal CD117^+^ mast cells in the CGRP + LL37-treated group, which was reduced after rimegepant or ritlecitinib treatment (**Figures 7G, H**). Western blotting analysis showed that both treatments suppressed JAK3 and STAT1 phosphorylation in lesional skin (**Figures 7I, J**). Concurrently, lesional skin levels of β-hexosaminidase, histamine, IL-6, TNF-α, CXCL10, CCL2, and VEGF were significantly decreased after either treatment (**Figure 7K**). Together, these findings indicate that pharmacological blockade of the CGRP receptor–JAK3–STAT1 axis alleviates CGRP-aggravated rosacea-like inflammation in mice.

## 4 Discussion

Rosacea is increasingly recognized as a disorder in which neurovascular dysregulation intersects with innate immune activation (51–53). In this context, neuropeptides released from sensory nerves may act as upstream signals that shape cutaneous inflammation and vascular reactivity. Among these mediators, CGRP has long been implicated in rosacea pathophysiology because of its potent vasodilatory activity, ability to evoke flushing, and association with neurogenic inflammation (13, 16, 25, 54). However, the cellular targets and intracellular pathways through which CGRP amplifies rosacea-associated inflammation remain incompletely understood. In the present study, we identify a CGRP-associated neuroimmune pathway in which CALCRL/RAMP1-linked JAK3–STAT1 signaling promotes mast cell-mediated inflammatory amplification in rosacea. By integrating transcriptomic analysis, clinical sample validation, tissue localization, *in vitro* mast-cell experiments, mast-cell depletion, and pharmacological intervention in rosacea-like mouse models, our findings provide mechanistic evidence linking CGRP-associated neuroimmune signaling to mast-cell activation in rosacea.

Previous studies have reported elevated serum CGRP levels in patients with rosacea, and both subcutaneous injection and intravenous administration of CGRP have been shown to provoke flushing (19, 55). In the present study, we confirmed that serum CGRP levels were elevated in rosacea and further showed that they positively correlated with flushing and erythema severity, but not with overall disease severity assessed by IGA. This pattern suggests that CGRP may be more closely related to neurovascular symptoms than to overall lesion burden. Consistent with this clinical association, lesional skin showed increased CGRP immunoreactivity and enhanced localization of the CGRP receptor components CALCRL and RAMP1 in dermal CD117^+^ mast cells. These findings support the concept that mast cells may function as cellular recipients of CGRP-associated neuroimmune signals in rosacea lesions.

Mast cells are strategically positioned at neurovascular–immune interfaces and are capable of translating neuronal signals into inflammatory and vasoactive responses (7, 8). Previous studies have shown that mast cells are required for LL37-induced rosacea-like inflammation, highlighting their importance in disease initiation and amplification (4, 56). In our study, CGRP alone did not produce overt rosacea-like dermatitis *in vivo* but markedly exacerbated LL37-induced erythema, dermal inflammatory infiltration, and mast-cell accumulation. The increased mast-cell numbers may reflect both local expansion and enhanced recruitment. CGRP has been reported to promote mast-cell proliferation, and our data showed that CGRP increased CCL2 secretion, which may facilitate the recruitment of circulating mast-cell progenitors via the CCL2–CCR2 axis (57–59). Importantly, ACK2-mediated mast-cell lineage depletion substantially attenuated the exacerbating effect of CGRP in the LL37 model. These findings suggest that CGRP functions primarily as an inflammatory amplifier in a primed cutaneous microenvironment rather than as an isolated inducer of rosacea-like inflammation, further supporting a functional role for mast cells in this setting. Notably, the biological consequences of CGRP-mediated neuroimmune signaling appear to be highly context dependent. In neurogenic inflammation, CGRP can promote vasodilation, mast-cell activation, and amplification of cutaneous inflammatory responses (7, 60, 61), whereas in other settings it may exert anti-inflammatory or tissue-protective effects, including roles in wound repair (62–64). These apparently divergent observations highlight the need to define cell-type- and disease-specific CGRP signaling mechanisms, particularly in the mast-cell compartment of rosacea lesions.

Current literature implicates a marked upregulation of JAK3-associated signaling in patients with papulopustular rosacea (27). STAT1-dependent transcription can shape the production of cytokines and chemokines that orchestrate leukocyte recruitment, including CCL2 and CXCL10, which are enriched in lesional skin (65, 66). Mechanistically, our data identify JAK3–STAT1 as a previously underrecognized signaling route downstream of CGRP receptor activation in mast cells. This finding does not exclude the involvement of the JAK2–STAT3 pathway previously reported by our group in rosacea (30). The JAK–STAT network is highly interconnected, with substantial overlap and crosstalk among different signaling branches. Therefore, the potential roles of other JAK–STAT pathways in CGRP-related rosacea inflammation warrant further investigation. In LUVA mast cells, CGRP stimulation increased CALCRL/RAMP1 expression, induced rapid phosphorylation of JAK3 and STAT1, and promoted β-hexosaminidase and histamine release together with inflammatory mediator production. Pharmacological blockade with rimegepant or ritlecitinib reduced JAK3–STAT1 phosphorylation, mast-cell degranulation, histamine release, and secretion of IL-6, TNF-α, CCL2, CXCL10, and VEGF. These mediators are highly relevant to rosacea pathogenesis: IL-6 and TNF-α amplify local inflammation, CCL2 and CXCL10 promote leukocyte recruitment, and VEGF contributes to vascular activation and remodeling. Thus, placing the CGRP–CALCRL/RAMP1 axis upstream of JAK3–STAT1 signaling provides a mechanistic framework linking neurogenic stimulation, mast-cell activation, inflammatory mediator production, and persistent erythema.

CGRP classically signals through the CALCRL/RAMP1 receptor complex to activate the Gαs–adenylyl cyclase–cAMP–PKA pathway (67). Consistent with this canonical signaling model, CGRP increased intracellular cAMP levels and PKA phosphorylation in LUVA mast cells. However, pharmacological dissection suggested that CGRP-induced JAK3–STAT1 activation was not primarily mediated by cAMP–PKA signaling. Suramin reduced CGRP-induced cAMP accumulation and PKA phosphorylation but did not abolish JAK3 or STAT1 phosphorylation, whereas forskolin enhanced cAMP production and PKA activation without independently inducing JAK3–STAT1 activation. These findings indicate that, in mast cells, CGRP can engage a non-canonical signaling route associated with JAK3–STAT1 activation. Functional inhibition further supports a functional contribution of each pathway to CGRP-induced mast cell degranulation. These findings do not exclude potential crosstalk between cAMP-dependent and JAK–STAT pathways under specific conditions; however, they suggest that CGRP-induced JAK3–STAT1 activation cannot be explained solely by classical cAMP–PKA activation.

The potential connection between CALCRL and JAK3 was further supported by protein–protein docking and reciprocal co-immunoprecipitation. Docking analysis predicted a favorable interaction interface between CALCRL and JAK3, and co-immunoprecipitation in an overexpression system supported a physical association between these proteins. These findings provide a plausible molecular basis for receptor-associated JAK3 activation after CGRP stimulation. However, the HEK293T overexpression system may not fully reflect the native receptor and signaling environment of human mast cells. Therefore, further investigation of the precise molecular mechanisms by which the CALCRL–RAMP1 receptor complex activates JAK3 in mast cells represents an important direction for future research.

The therapeutic implications of this pathway are supported by the *in vivo* pharmacological intervention experiments. Both rimegepant, a CGRP receptor antagonist, and ritlecitinib, a JAK3 inhibitor, alleviated CGRP-aggravated LL37-induced rosacea-like dermatitis. These treatments reduced erythema, dermal inflammatory infiltration, mast-cell accumulation, JAK3–STAT1 phosphorylation, and inflammatory mediator production in lesional skin. These results suggest that targeting either the upstream CGRP receptor or the downstream JAK3 signaling node may suppress mast cell-mediated neuroimmune amplification. Given the established efficacy of CGRP receptor antagonists in migraine and the growing clinical experience with JAK inhibitors in inflammatory skin diseases (14, 68, 69), these findings provide proof-of-concept evidence that the CGRP receptor–JAK3–STAT1 axis may represent a mechanism-based therapeutic target, particularly for patients with neurovascular-dominant or treatment-refractory disease.

Several limitations should be acknowledged. First, the clinical sample size, especially for lesional skin biopsies, was limited, and larger cohorts are needed to validate the association between this pathway and specific rosacea phenotypes. Second, the present study primarily focused on exogenous CGRP exposure, and the contribution of endogenous CGRP, including CGRP from neuronal and non-neuronal sources, remains to be determined. In particular, whether LL37-induced endogenous CGRP contributes to inflammatory changes or activation of the CALCRL/RAMP1–JAK3–STAT1 axis requires further investigation. Finally, the mast-cell depletion and pharmacological intervention experiments were performed in different mouse strains and only female animals were used; therefore, potential strain- and sex-related influences cannot be fully excluded. These limitations highlight important directions for future investigation.

**Figure 8:**
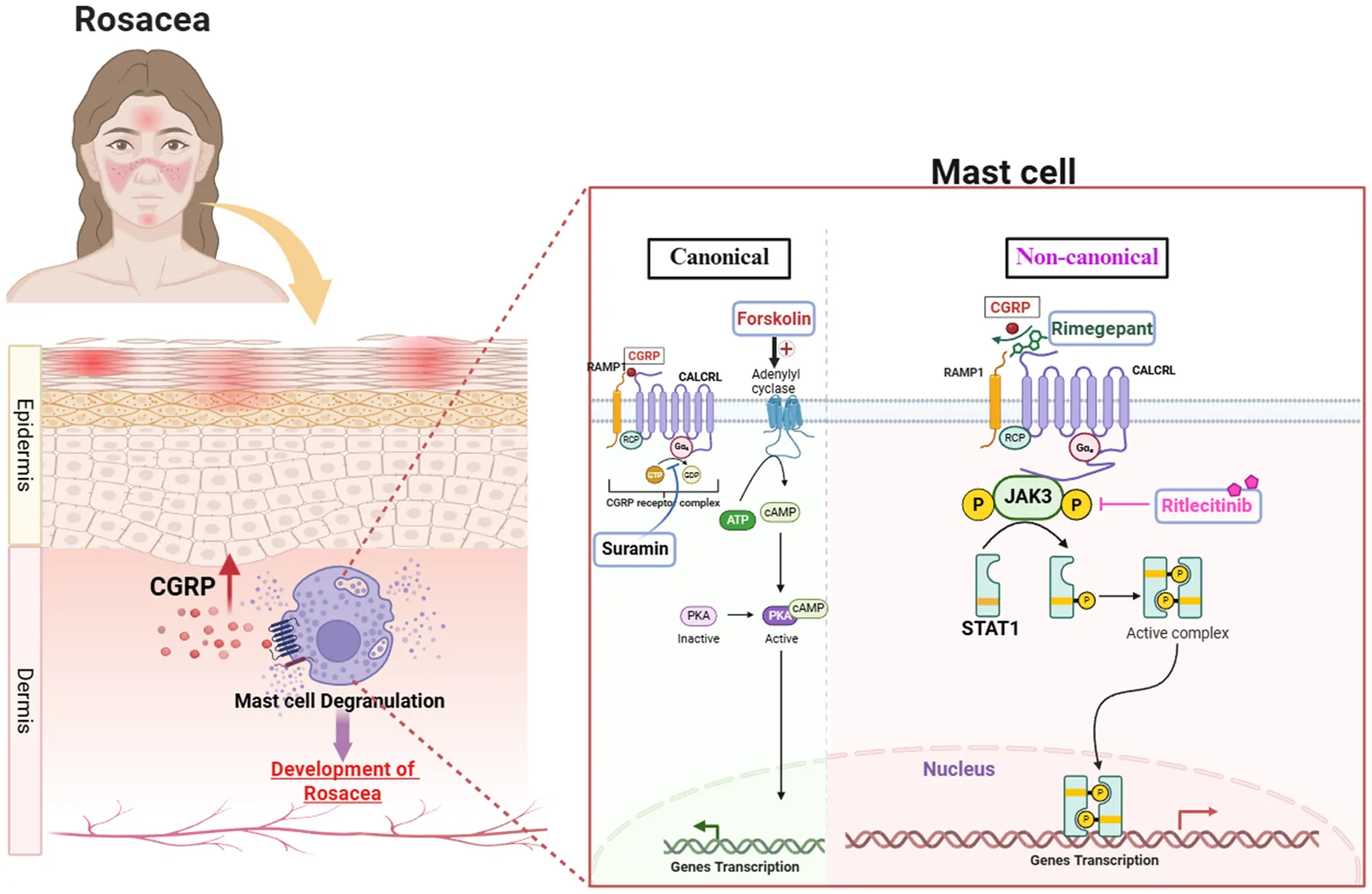
Schematic model of CGRP-driven mast-cell activation via the CALCRL/RAMP1–JAK3–STAT1 axis in rosacea. Elevated CGRP in rosacea activates mast cells through the CALCRL/RAMP1 receptor complex. Although CGRP can trigger the canonical Gαs–cAMP–PKA pathway, the present findings highlight a non-canonical JAK3–STAT1 signaling route linking CGRP stimulation to mast-cell degranulation and inflammatory mediator production. Blocking CGRP receptor signaling with rimegepant or inhibiting JAK3 with ritlecitinib attenuates this response, suggesting that the CALCRL/RAMP1–JAK3–STAT1 axis may represent a potential therapeutic target in rosacea.

In summary, this study identifies a CGRP-associated neuroimmune axis in rosacea, in which CALCRL/RAMP1-linked JAK3–STAT1 signaling contributes to mast-cell activation and inflammatory amplification through a mechanism that is not primarily mediated by the canonical cAMP–PKA pathway (**Figure 8**). By connecting CGRP signaling with mast cell-mediated innate immune activation, this pathway provides a mechanistic framework for understanding neurovascular instability, persistent erythema, and inflammatory amplification in rosacea. These findings expand current understanding of rosacea pathogenesis and suggest that the CGRP–CALCRL/RAMP1–JAK3–STAT1 axis may represent a potential target for future therapeutic investigation.

## Data Availability

The datasets presented in this study can be found in online repositories. The names of the repository/repositories and accession number(s) can be found in the article/**Supplementary Material**.
