# Supplementary material for: Calcitonin gene-related peptide promotes mast cell-mediated neuroimmune inflammation through the CALCRL/RAMP1–JAK3–STAT1 axis in rosacea
Source: Front Immunol. 2026 Sep 2;17:1930591. doi: 10.3389/fimmu.2026.1930591 (PMC13579260; doi:10.3389/fimmu.2026.1930591)
Supplement: Supplementary file 1 [file SupplementaryFile2.pdf]

frontiers

The International Society for the Advancement of Cytometry (ISAC) has highlighted the importance of including comprehensive methodological information to ensure data reproducibility and reliability. In line with this, Frontiers in Immunology now requires authors to submit a checklist for manuscripts that involve flow or mass cytometry. This checklist helps standardize the reporting process, improving the quality and transparency of published data. By doing so, we support scientific progress, making it easier for other researchers to replicate and validate experiments.

This form should be submitted with any manuscripts using flow or mass cytometry.

Sample/specimen/material description

☐ Total blood

☐ PBMCs

☐ Organ digests

Other Bone marrow single-cell suspensions

Did the samples suffer any treatment before or after incubation with the antibodies?

☐ Drug ___

☐ Cell permeabilization ___

☐ Dye ___

☐ Propidium iodine

☐ Not applicable

Other ACK Lysing Buffer (Solarbio, R1013) and Fc receptor blocking with anti-CD16/CD32 antibody (Invitrogen, 14-0161-82) before staining

Instrument and antibodies

Name of the Cytometer Mindray BriCyte MX flow cytometer

Antibodies and targets	Fluorochrome/ Metal	Catalog number/Company
e.g. anti-CD4	FITC	Cat. XXX/ XXX ltd.
anti-CD45	FITC	Cat.11-0451-82/Invitrogen

anti-CD117	APC	Cat.17-1171-82/Invitrogen
anti-integrin β7	PE	Cat.12-5867-42/Invitrogen

Data analyses

1. Name of the software FlowJo software (version 10.8.1.)_____________________________________
2. Reference gating strategy in the manuscript or supplementary material

Gating strategy in (eg Figure X) Figure S5A________________________________
