# Supplementary material for: Calcitonin gene-related peptide promotes mast cell-mediated neuroimmune inflammation through the CALCRL/RAMP1–JAK3–STAT1 axis in rosacea
Source: Front Immunol. 2026 Sep 2;17:1930591. doi: 10.3389/fimmu.2026.1930591 (PMC13579260; doi:10.3389/fimmu.2026.1930591)
Supplement: Supplementary file 2 [file SupplementaryFile1.docx]

**Supplementary Figure**

**Supplementary Figure 1. Quantification of CALCRL, RAMP1, p-JAK3, and p-STAT1 expression in rosacea lesional skin**

**(A–D)** Quantification of the relative fluorescence intensities of CALCRL, RAMP1, p-JAK3, and p-STAT1 in healthy skin (HS) and rosacea lesional skin, corresponding to the immunofluorescence staining shown in (Figure 3E–H) (*n* = 5). Data are presented as mean ± SD. Statistical significance was determined using an unpaired two-tailed Student’s *t*-test. ***P* < 0.01, ****P* < 0.001.

**Supplementary Figure 2.** **Effects of CGRP on LUVA cell viability and β-hexosaminidase release.**

**(A)** LUVA cells were treated with CGRP at the indicated concentrations for 0, 24, 48, and 72 h, and cell viability was assessed using the CCK-8 assay (*n* = 3). **(B)** β-hexosaminidase release from LUVA cells after stimulation with 0–200 nM CGRP for 30 min (*n* = 3). Data are presented as mean ± SD. Statistical significance was determined using two-way ANOVA followed by Dunnett’s multiple-comparisons test for **(A)** and one-way ANOVA followed by Dunnett’s multiple-comparisons test for **(B)**, with each CGRP-treated group compared with the corresponding control group. ***P* < 0.01, ****P* < 0.001, *****P* < 0.0001.

**Supplementary Figure 3.** **CGRP activates the cAMP–PKA signaling pathway in LUVA cells.**

**(A)** Intracellular cAMP levels in LUVA cells following CGRP stimulation were measured by ELISA (*n* = 6). **(B)** Western blotting analysis showing p-PKA, PKA, and α-tubulin levels in LUVA cells stimulated with CGRP for 0, 15, 30, and 45 min. **(C)** Quantification of western blotting results. p-PKA levels were normalized to total PKA, and total PKA levels were normalized to α-tubulin. Western blotting experiments were independently repeated three times. Data are presented as mean ± SD. Statistical significance was determined using an unpaired two-tailed Student’s *t*-test for **(A)** and one-way ANOVA for **(C)**. ****P* < 0.001, *****P* < 0.0001.

**Supplementary Figure 4.** **CGRP activates JAK3–STAT1 independently of the canonical cAMP–PKA pathway in LUVA mast cells**

**(A)** LUVA cells were treated with suramin at the indicated concentrations for 6, 12, or 24 h, and cell viability was assessed using the CCK-8 assay (*n* = 3). **(B)** Intracellular cAMP levels in LUVA cells treated with CGRP in the presence or absence of suramin were measured by ELISA (*n* = 9). **(C, D)** Western blotting analysis **(C)** and quantification **(D)** of p-JAK3, JAK3, p-STAT1, STAT1, p-PKA, and PKA levels in LUVA cells treated with CGRP in the presence or absence of suramin. **(E)** LUVA cells were treated with forskolin at the indicated concentrations for 6, 12, or 24 h, and cell viability was assessed using the CCK-8 assay (*n* = 3). **(F)** Intracellular cAMP levels in LUVA cells stimulated with CGRP in the presence or absence of forskolin were measured by ELISA (*n* = 9). **(G, H)** Western blotting analysis **(G)** and quantification **(H)** of p-JAK3, JAK3, p-STAT1, STAT1, p-PKA, and PKA levels in LUVA cells treated with CGRP in the presence or absence of forskolin. Western blotting experiments were independently repeated three times. p-JAK3, p-STAT1, and p-PKA levels were normalized to total JAK3, STAT1, and PKA, respectively, whereas total JAK3, STAT1, and PKA levels were normalized to α-tubulin. **(I)** LUVA cells were treated with fludarabine at the indicated concentrations for 6, 12, or 24 h, and cell viability was assessed using the CCK-8 assay (*n* = 3). **(J, K)** β-hexosaminidase release **(J)** and histamine release **(K)** from CGRP-stimulated LUVA cells following treatment with suramin, fludarabine, or their combination. Data are presented as mean ± SD. Statistical significance in **(A), (E)** and **(I)** was determined using two-way ANOVA followed by Dunnett’s multiple-comparisons test, comparing each treatment group with the corresponding control group at the same time point. Statistical significance in **(B)**, **(D)**, **(F)**, **(H), (J)** and **(K)** was determined using one-way ANOVA followed by Tukey’s multiple-comparisons test for the indicated comparisons. ns, not significant; **P* < 0.05, ***P* < 0.01, ****P* < 0.001, *****P* < 0.0001.

**Supplementary Figure 5. Validation of ACK2-mediated mast-cell lineage depletion.**

**(A)** Representative flow-cytometry gating strategy used to identify bone marrow CD45⁺SSC^low^CD117⁺integrin β7⁺ mast cell progenitor-enriched cells in control and ACK2-treated mice. **(B)** Representative toluidine blue staining images and quantification of dermal mast cells in control and ACK2-treated mice. Data are presented as mean ± SEM; *n* = 5 mice per group. Statistical significance was determined using a two-tailed unpaired Student’s *t*-test. **P* < 0.05.
